# 1-[6-(3,5-Di­methyl­pyrazol-1-yl)-1,2,4,5-tetra­zin-3-yl]guanidin-2-ium perchlorate methanol monosolvate

**DOI:** 10.1107/S1600536813020448

**Published:** 2013-07-27

**Authors:** Yong-Peng Hu, Biao Yan, Jie Li, Hai-Xia Ma

**Affiliations:** a School of Chemical Engineering, Northwest University, Xi’an 710069, Shaanxi, People’s Republic of China; b School of Chemistry and Chemical Engineering, Yulin University, Yulin 719000, Shaanxi, People’s Republic of China

## Abstract

In the title solvated salt, C_8_H_12_N_9_^+^·ClO_4_^−^·CH_3_OH, the dihedral angle between the tetra­zine and pyrazole rings is 26.05 (7)°. The two N atoms bonded to the 1,2,4,5-tetra­zine ring deviate from the plane defined by its four N atoms by 0.234 (2) and 0.186 (2) Å. There is an intra­molecular N—H⋯N hydrogen bond between the protonated guanidine fragment and one of the tetra­zine N atoms. In the crystal, two cations and two perchlorate anions are connected *via* N—H⋯O hydrogen bonds into centrosymmetric assemblies. These assemblies are further linked into a two-dimensional network parallel to (100) *via* bifurcated O—H⋯(N,N) hydrogen bonds formed with the bridging methanol mol­ecules.

## Related literature

For 1,2,4,5-tetra­zine heterocycles containing strained ring systems, see: Boger & Zhang (1991 ▶); Chavez *et al.* (2004 ▶); Saikia *et al.* (2009 ▶).
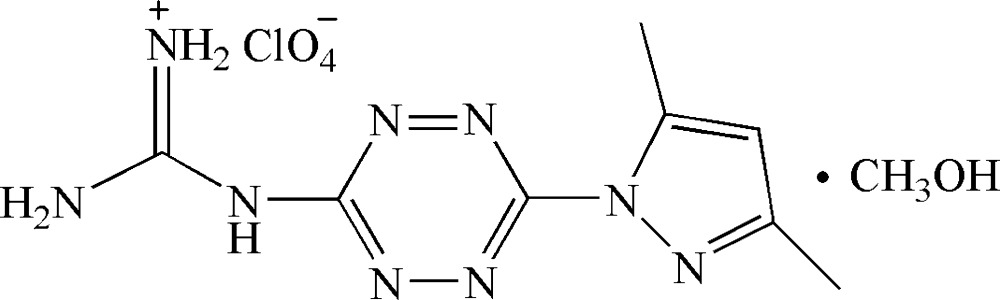


## Experimental

### 

#### Crystal data

C_8_H_12_N_9_^+^·ClO_4_^−^·CH_4_O
*M* *_r_* = 365.76
Monoclinic, 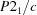

*a* = 12.7906 (15) Å
*b* = 8.0149 (10) Å
*c* = 16.644 (2) Å
β = 108.305 (1)°
*V* = 1619.9 (3) Å^3^
*Z* = 4
Mo *K*α radiation
μ = 0.28 mm^−1^
*T* = 296 K
0.38 × 0.28 × 0.19 mm

#### Data collection

Bruker SMART APEXII CCD area-detector diffractometer
Absorption correction: multi-scan (*SADABS*; Sheldrick, 2000 ▶) *T*_min_ = 0.902, *T*_max_ = 0.948
7710 measured reflections
2875 independent reflections
2426 reflections with *I* > 2σ(*I*)
*R*_int_ = 0.022

#### Refinement

*R*[*F* ^2^ > 2σ(*F* ^2^)] = 0.038
*wR*(*F* ^2^) = 0.113
*S* = 1.06
2875 reflections
222 parameters
H-atom parameters constrained
Δρ_max_ = 0.24 e Å^−3^
Δρ_min_ = −0.27 e Å^−3^

### 

Data collection: *APEX2* (Bruker, 2003 ▶); cell refinement: *SAINT* (Bruker, 2003 ▶); data reduction: *SAINT*; program(s) used to solve structure: *SHELXS97* (Sheldrick, 2008 ▶); program(s) used to refine structure: *SHELXL97* (Sheldrick, 2008 ▶); molecular graphics: *SHELXTL* (Sheldrick, 2008 ▶); software used to prepare material for publication: *SHELXTL*.

## Supplementary Material

Crystal structure: contains datablock(s) I. DOI: 10.1107/S1600536813020448/gk2585sup1.cif

Structure factors: contains datablock(s) I. DOI: 10.1107/S1600536813020448/gk2585Isup2.hkl

Supplementary material file. DOI: 10.1107/S1600536813020448/gk2585Isup3.cml

Additional supplementary materials:  crystallographic information; 3D view; checkCIF report

## Figures and Tables

**Table 1:** Hydrogen-bond geometry (Å, °)

*D*—H⋯*A*	*D*—H	H⋯*A*	*D*⋯*A*	*D*—H⋯*A*
N1—H1*A*⋯O2^i^	0.86	2.54	3.101 (3)	124
O5—H5⋯N9^ii^	0.82	2.05	2.866 (2)	173
O5—H5⋯N5^ii^	0.82	2.50	2.940 (2)	114
N2—H2*A*⋯O3^iii^	0.86	2.50	3.251 (3)	146
N2—H2*A*⋯O2^iii^	0.86	2.37	3.118 (3)	146
N1—H1*A*⋯O2^iii^	0.86	2.37	3.120 (3)	146
N3—H3⋯O5	0.86	1.90	2.700 (2)	153
N2—H2*B*⋯N7	0.86	2.09	2.713 (2)	129
N1—H1*B*⋯O5	0.86	2.37	3.085 (3)	140

Symmetry codes: (i) 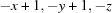; (ii) 
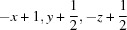
; (iii) 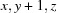.

**Fig. 1.**
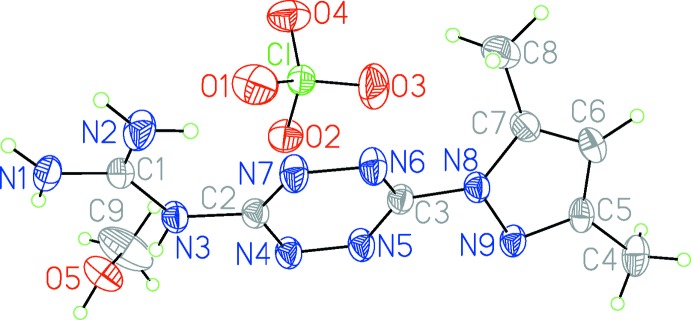
The asymmetric unit of the title compound with the atom-numbering scheme. Displacement ellipsoids are drawn at the 30% probability level and H atoms are drawn as spheres of arbitrary radius.

**Fig. 2.**
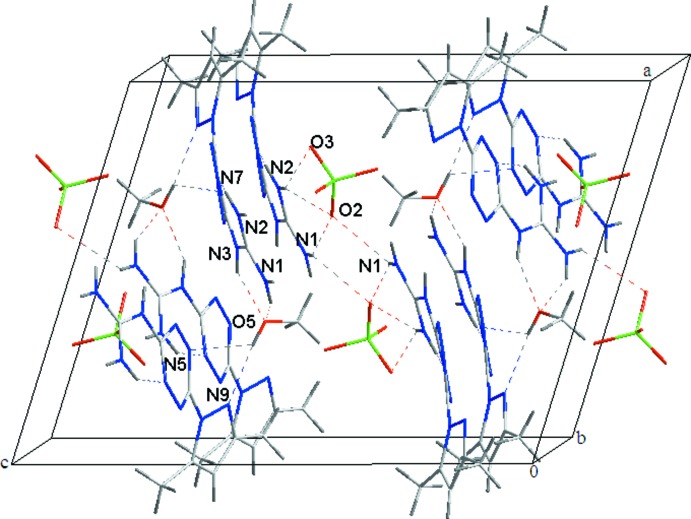
Crystal packing diagram. Hydrogen bonds are shown with dashed lines.

## supplementary crystallographic information

### Comment

Heterocycles with high nitrogen and low carbon content that are free of halogens possess desirable stability. Recently, considerable attention has been paid to 1,2,4,5-tetrazine heterocycles containing strained ring systems (Boger and Zhang, 1991; Chavez *et al.*, 2004; Saikia *et al.*, 2009). This makes them good candidates for energetic materials (propellants or explosives). Heteroatom substituted tetrazine derivatives such as 3,6-diguanidino-1,2,4,5-tetrazine (DGTz) (Chavez *et al.*, 2004) and 3,6-bis(1*H*-1,2,3,4-tetrazol-5-ylamino)-1,2,4,5-tetrazine (BTATz) (Saikia *et al.*, 2009) are readily accessible from 3,6-bis(3,5-dimethylpyrazol-1-yl)-1,2,4,5-tetrazine (BT). 3-Guanidyl-6-(3,5-dimethylpyrazol-1-yl)-1,2,4,5-tetrazine (GDPTz) also is a derivative of BT and we report here the crystal structure of its perchlorate salt methanol monosolvate..

### Experimental

Methanol (100 ml), guanidinium nitrate (11.8 g,0.098 mol) and sodium methoxide (4.4 g 0.098 mol) were stirred for 45 minutes. 3,6-Bis(3,5-dimethylpyrazol-1-yl)-1,2,4,5-tetrazine (12.4 g, 46 mmol) was added in one portion and stirred at room temperature for 12 h. The dark red slurry, composed mainly of DGTz mixed with a small amount of GDPTz, was filtered and washed with amounts of copious water and transferred to a 500 ml beaker. The solids were suspended in water (200 ml) and 70% perchloric acid (32 ml) was added with stirring; the suspension slowly turned into an orange solution. Orange needles precipitate was gained after a few minutes of stirring. The slurry was heated to re-dissolve the precipitate and cooled to room temperature and then placed in the refrigerator for several hours. The orange needles were collected by filtration, the filtrate was concentrated *in vacuo*, the solid product was washed with ethanol and purified by recrystallization from methanol to give the pure saffron compound in 4.1% yield. Crystals were obtained from methanol, by slow evaporation at room temperature. Elemental analysis calculated for C_9_H_16_N_9_O_5_Cl: C 29.56, N 34.47, H 4.41%; found: C 29.19, N 34.10, H 4.60%.

### Refinement

H atoms were placed at calculated idealized positions and refined using a riding model, with C—H distances in the range 0.93–0.96 Å, N—H distance 0.86 Å, and O—H distance 0.82 Å.

### Crystal data

**Table:**

C_8_H_12_N_9_^+^·ClO_4_^−^·CH_4_O	*F*(000) = 760
*M**_r_* = 365.76	*D*_x_ = 1.500 Mg m^−^^3^
Monoclinic, *P*2_1_/*c*	Mo *K*α radiation, λ = 0.71073 Å
Hall symbol: -P 2ybc	Cell parameters from 3433 reflections
*a* = 12.7906 (15) Å	θ = 2.6–25.8°
*b* = 8.0149 (10) Å	µ = 0.28 mm^−^^1^
*c* = 16.644 (2) Å	*T* = 296 K
β = 108.305 (1)°	Block, yellow
*V* = 1619.9 (3) Å^3^	0.38 × 0.28 × 0.19 mm
*Z* = 4	

### Data collection

**Table:**

Bruker SMART APEXII CCD area-detector diffractometer	2875 independent reflections
Radiation source: fine-focus sealed tube	2426 reflections with *I* > 2σ(*I*)
Graphite monochromator	*R*_int_ = 0.022
phi and ω scans	θ_max_ = 25.1°, θ_min_ = 2.6°
Absorption correction: multi-scan (*SADABS*; Sheldrick, 2000)	*h* = −14→15
*T*_min_ = 0.902, *T*_max_ = 0.948	*k* = −9→6
7710 measured reflections	*l* = −19→18

### Refinement

**Table:**

Refinement on *F*^2^	Secondary atom site location: difference Fourier map
Least-squares matrix: full	Hydrogen site location: inferred from neighbouring sites
*R*[*F*^2^ > 2σ(*F*^2^)] = 0.038	H-atom parameters constrained
*wR*(*F*^2^) = 0.113	*w* = 1/[σ^2^(*F*_o_^2^) + (0.0557*P*)^2^ + 0.5826*P*] where *P* = (*F*_o_^2^ + 2*F*_c_^2^)/3
*S* = 1.06	(Δ/σ)_max_ < 0.001
2875 reflections	Δρ_max_ = 0.24 e Å^−^^3^
222 parameters	Δρ_min_ = −0.27 e Å^−^^3^
0 restraints	Extinction correction: *SHELXL97* (Sheldrick, 2008), Fc^*^=kFc[1+0.001xFc^2^λ^3^/sin(2θ)]^-1/4^
Primary atom site location: structure-invariant direct methods	Extinction coefficient: 0.0149 (15)

### Special details

**Table:**

Geometry. All e.s.d.'s (except the e.s.d. in the dihedral angle between two l.s. planes) are estimated using the full covariance matrix. The cell e.s.d.'s are taken into account individually in the estimation of e.s.d.'s in distances, angles and torsion angles; correlations between e.s.d.'s in cell parameters are only used when they are defined by crystal symmetry. An approximate (isotropic) treatment of cell e.s.d.'s is used for estimating e.s.d.'s involving l.s. planes.
Refinement. Refinement of *F*^2^ against ALL reflections. The weighted *R*-factor *wR* and goodness of fit *S* are based on *F*^2^, conventional *R*-factors *R* are based on *F*, with *F* set to zero for negative *F*^2^. The threshold expression of *F*^2^ > σ(*F*^2^) is used only for calculating *R*-factors(gt) *etc*. and is not relevant to the choice of reflections for refinement. *R*-factors based on *F*^2^ are statistically about twice as large as those based on *F*, and *R*- factors based on ALL data will be even larger.

### Fractional atomic coordinates and isotropic or equivalent isotropic displacement parameters (Å^2^)

**Table:**

	*x*	*y*	*z*	*U*_iso_*/*U*_eq_	
Cl	0.71723 (4)	0.14781 (6)	0.07027 (3)	0.0510 (2)	
N2	0.67200 (16)	0.7743 (2)	0.19606 (13)	0.0613 (5)	
H2A	0.6775	0.8725	0.1769	0.074*	
H2B	0.7265	0.7322	0.2353	0.074*	
N3	0.56882 (13)	0.5343 (2)	0.19387 (11)	0.0463 (4)	
H3	0.5025	0.4971	0.1807	0.056*	
N1	0.49649 (15)	0.7504 (3)	0.10588 (13)	0.0639 (6)	
H1A	0.5002	0.8483	0.0858	0.077*	
H1B	0.4373	0.6923	0.0868	0.077*	
N4	0.61470 (13)	0.2743 (2)	0.24859 (12)	0.0503 (4)	
N5	0.68994 (14)	0.1634 (2)	0.28457 (12)	0.0524 (5)	
N6	0.82827 (13)	0.3725 (2)	0.31430 (11)	0.0510 (4)	
N7	0.75184 (14)	0.4852 (2)	0.27682 (11)	0.0516 (4)	
N9	0.84660 (13)	−0.0495 (2)	0.37776 (10)	0.0468 (4)	
C5	0.93968 (17)	−0.1338 (2)	0.40593 (13)	0.0473 (5)	
C6	1.02667 (17)	−0.0472 (3)	0.39046 (14)	0.0544 (5)	
H6	1.0995	−0.0824	0.4048	0.065*	
C7	0.98545 (16)	0.0966 (3)	0.35103 (13)	0.0513 (5)	
N8	0.87506 (13)	0.0947 (2)	0.34434 (10)	0.0445 (4)	
O1	0.69859 (19)	0.3153 (2)	0.08793 (16)	0.0991 (7)	
O2	0.61828 (13)	0.0547 (2)	0.05927 (12)	0.0742 (5)	
O3	0.80093 (16)	0.0795 (3)	0.13929 (14)	0.1041 (7)	
O4	0.74576 (18)	0.1425 (3)	−0.00434 (12)	0.0920 (7)	
O5	0.36726 (13)	0.4239 (2)	0.10212 (12)	0.0690 (5)	
H5	0.3085	0.4369	0.1114	0.103*	
C1	0.58130 (17)	0.6897 (2)	0.16581 (13)	0.0455 (5)	
C2	0.65001 (15)	0.4300 (2)	0.24091 (12)	0.0421 (4)	
C3	0.79545 (15)	0.2160 (2)	0.31182 (12)	0.0425 (4)	
C4	0.9443 (2)	−0.3002 (3)	0.44739 (16)	0.0669 (7)	
H4A	0.8769	−0.3192	0.4598	0.100*	
H4B	1.0050	−0.3024	0.4990	0.100*	
H4C	0.9540	−0.3858	0.4100	0.100*	
C8	1.0389 (2)	0.2301 (4)	0.3155 (2)	0.0821 (9)	
H8A	1.0451	0.3296	0.3488	0.123*	
H8B	0.9950	0.2528	0.2582	0.123*	
H8C	1.1109	0.1941	0.3166	0.123*	
C9	0.3592 (3)	0.2881 (5)	0.0482 (3)	0.1234 (15)	
H9A	0.4295	0.2681	0.0405	0.185*	
H9B	0.3056	0.3116	−0.0056	0.185*	
H9C	0.3371	0.1910	0.0725	0.185*	

### Atomic displacement parameters (Å^2^)

**Table:**

	*U*^11^	*U*^22^	*U*^33^	*U*^12^	*U*^13^	*U*^23^
Cl	0.0464 (3)	0.0461 (3)	0.0566 (3)	−0.0045 (2)	0.0103 (2)	0.0036 (2)
N2	0.0621 (12)	0.0374 (10)	0.0784 (13)	−0.0025 (9)	0.0135 (10)	0.0134 (9)
N3	0.0380 (8)	0.0378 (9)	0.0616 (10)	0.0032 (7)	0.0136 (8)	0.0093 (8)
N1	0.0550 (11)	0.0606 (12)	0.0739 (13)	0.0101 (9)	0.0171 (9)	0.0296 (10)
N4	0.0410 (9)	0.0407 (9)	0.0675 (11)	0.0020 (7)	0.0144 (8)	0.0137 (8)
N5	0.0423 (9)	0.0399 (9)	0.0713 (12)	0.0019 (7)	0.0127 (8)	0.0140 (8)
N6	0.0429 (9)	0.0379 (9)	0.0628 (11)	0.0010 (7)	0.0033 (8)	0.0040 (8)
N7	0.0481 (10)	0.0351 (9)	0.0636 (11)	0.0008 (7)	0.0058 (8)	0.0042 (8)
N9	0.0450 (9)	0.0371 (9)	0.0537 (10)	0.0021 (7)	0.0091 (7)	0.0080 (7)
C5	0.0508 (11)	0.0380 (11)	0.0452 (11)	0.0100 (9)	0.0039 (9)	−0.0030 (8)
C6	0.0427 (11)	0.0534 (13)	0.0634 (13)	0.0130 (10)	0.0111 (10)	−0.0024 (10)
C7	0.0421 (11)	0.0551 (13)	0.0572 (12)	0.0039 (10)	0.0166 (9)	0.0007 (10)
N8	0.0400 (9)	0.0385 (9)	0.0524 (10)	0.0041 (7)	0.0109 (7)	0.0076 (7)
O1	0.1156 (17)	0.0471 (10)	0.152 (2)	−0.0112 (11)	0.0675 (16)	−0.0110 (12)
O2	0.0597 (10)	0.0659 (11)	0.0934 (13)	−0.0216 (9)	0.0188 (9)	0.0095 (9)
O3	0.0709 (12)	0.1261 (19)	0.0903 (14)	0.0117 (13)	−0.0106 (10)	0.0277 (13)
O4	0.0944 (14)	0.1182 (18)	0.0745 (12)	−0.0142 (13)	0.0424 (11)	−0.0052 (12)
O5	0.0487 (9)	0.0724 (11)	0.0892 (12)	−0.0052 (8)	0.0265 (8)	−0.0270 (9)
C1	0.0468 (11)	0.0395 (11)	0.0528 (11)	0.0085 (9)	0.0197 (9)	0.0077 (9)
C2	0.0407 (10)	0.0365 (10)	0.0489 (11)	0.0022 (8)	0.0138 (8)	0.0040 (8)
C3	0.0408 (10)	0.0387 (10)	0.0457 (10)	0.0020 (8)	0.0104 (8)	0.0064 (8)
C4	0.0700 (15)	0.0437 (12)	0.0730 (16)	0.0112 (11)	0.0026 (12)	0.0095 (11)
C8	0.0601 (15)	0.0845 (19)	0.113 (2)	0.0046 (14)	0.0429 (15)	0.0253 (17)
C9	0.090 (2)	0.142 (3)	0.149 (3)	−0.019 (2)	0.053 (2)	−0.089 (3)

### Geometric parameters (Å, º)

**Table:**

Cl—O4	1.4007 (19)	N9—N8	1.381 (2)
Cl—O1	1.410 (2)	C5—C6	1.402 (3)
Cl—O3	1.4118 (19)	C5—C4	1.494 (3)
Cl—O2	1.4307 (16)	C6—C7	1.349 (3)
N2—C1	1.301 (3)	C6—H6	0.9300
N2—H2A	0.8600	C7—N8	1.381 (2)
N2—H2B	0.8600	C7—C8	1.489 (3)
N3—C1	1.357 (3)	N8—C3	1.388 (2)
N3—C2	1.371 (2)	O5—C9	1.394 (3)
N3—H3	0.8600	O5—H5	0.8200
N1—C1	1.315 (3)	C4—H4A	0.9600
N1—H1A	0.8600	C4—H4B	0.9600
N1—H1B	0.8600	C4—H4C	0.9600
N4—N5	1.309 (2)	C8—H8A	0.9600
N4—C2	1.346 (3)	C8—H8B	0.9600
N5—C3	1.349 (3)	C8—H8C	0.9600
N6—C3	1.320 (3)	C9—H9A	0.9600
N6—N7	1.333 (2)	C9—H9B	0.9600
N7—C2	1.327 (2)	C9—H9C	0.9600
N9—C5	1.320 (2)		
			
O4—Cl—O1	108.79 (14)	N9—N8—C3	119.30 (15)
O4—Cl—O3	111.56 (14)	C7—N8—C3	129.05 (17)
O1—Cl—O3	109.53 (16)	C9—O5—H5	109.5
O4—Cl—O2	109.68 (12)	N2—C1—N1	121.56 (19)
O1—Cl—O2	108.81 (12)	N2—C1—N3	122.16 (19)
O3—Cl—O2	108.42 (13)	N1—C1—N3	116.28 (19)
C1—N2—H2A	120.0	N7—C2—N4	125.26 (17)
C1—N2—H2B	120.0	N7—C2—N3	120.94 (17)
H2A—N2—H2B	120.0	N4—C2—N3	113.76 (16)
C1—N3—C2	127.33 (17)	N6—C3—N5	125.62 (18)
C1—N3—H3	116.3	N6—C3—N8	117.78 (17)
C2—N3—H3	116.3	N5—C3—N8	116.49 (17)
C1—N1—H1A	120.0	C5—C4—H4A	109.5
C1—N1—H1B	120.0	C5—C4—H4B	109.5
H1A—N1—H1B	120.0	H4A—C4—H4B	109.5
N5—N4—C2	116.91 (16)	C5—C4—H4C	109.5
N4—N5—C3	117.09 (16)	H4A—C4—H4C	109.5
C3—N6—N7	116.75 (16)	H4B—C4—H4C	109.5
C2—N7—N6	117.18 (16)	C7—C8—H8A	109.5
C5—N9—N8	104.42 (16)	C7—C8—H8B	109.5
N9—C5—C6	111.15 (18)	H8A—C8—H8B	109.5
N9—C5—C4	121.4 (2)	C7—C8—H8C	109.5
C6—C5—C4	127.4 (2)	H8A—C8—H8C	109.5
C7—C6—C5	107.51 (18)	H8B—C8—H8C	109.5
C7—C6—H6	126.2	O5—C9—H9A	109.5
C5—C6—H6	126.2	O5—C9—H9B	109.5
C6—C7—N8	105.28 (19)	H9A—C9—H9B	109.5
C6—C7—C8	130.5 (2)	O5—C9—H9C	109.5
N8—C7—C8	124.1 (2)	H9A—C9—H9C	109.5
N9—N8—C7	111.63 (16)	H9B—C9—H9C	109.5
			
C2—N4—N5—C3	1.1 (3)	C2—N3—C1—N1	164.5 (2)
C3—N6—N7—C2	0.3 (3)	N6—N7—C2—N4	9.1 (3)
N8—N9—C5—C6	−0.6 (2)	N6—N7—C2—N3	−173.48 (18)
N8—N9—C5—C4	−179.98 (19)	N5—N4—C2—N7	−9.8 (3)
N9—C5—C6—C7	0.0 (3)	N5—N4—C2—N3	172.61 (18)
C4—C5—C6—C7	179.3 (2)	C1—N3—C2—N7	11.8 (3)
C5—C6—C7—N8	0.6 (2)	C1—N3—C2—N4	−170.56 (19)
C5—C6—C7—C8	−175.8 (3)	N7—N6—C3—N5	−9.0 (3)
C5—N9—N8—C7	1.0 (2)	N7—N6—C3—N8	174.88 (17)
C5—N9—N8—C3	−177.43 (17)	N4—N5—C3—N6	8.3 (3)
C6—C7—N8—N9	−1.0 (2)	N4—N5—C3—N8	−175.54 (17)
C8—C7—N8—N9	175.7 (2)	N9—N8—C3—N6	151.36 (18)
C6—C7—N8—C3	177.2 (2)	C7—N8—C3—N6	−26.8 (3)
C8—C7—N8—C3	−6.0 (4)	N9—N8—C3—N5	−25.1 (3)
C2—N3—C1—N2	−15.4 (3)	C7—N8—C3—N5	156.8 (2)

### Hydrogen-bond geometry (Å, º)

**Table:**

*D*—H···*A*	*D*—H	H···*A*	*D*···*A*	*D*—H···*A*
N1—H1*A*···O2^i^	0.86	2.54	3.101 (3)	124
O5—H5···N9^ii^	0.82	2.05	2.866 (2)	173
O5—H5···N5^ii^	0.82	2.50	2.940 (2)	114
N2—H2*A*···O3^iii^	0.86	2.50	3.251 (3)	146
N2—H2*A*···O2^iii^	0.86	2.37	3.118 (3)	146
N1—H1*A*···O2^iii^	0.86	2.37	3.120 (3)	146
N3—H3···O5	0.86	1.90	2.700 (2)	153
N2—H2*B*···N7	0.86	2.09	2.713 (2)	129
N1—H1*B*···O5	0.86	2.37	3.085 (3)	140

Symmetry codes: (i) −*x*+1, −*y*+1, −*z*; (ii) −*x*+1, *y*+1/2, −*z*+1/2; (iii) *x*, *y*+1, *z*.
